# 

**Published:** 1891-08-08

**Authors:** 


### NOTICE TO CORRESPONDENTS.

All MS., Letters, Books for Review, and other matters intended for the Editor should be addressed The Editor, The Lodge, Porchester Square, London, W.

The Editor cannot undertake to return rejected M.S., even when accompanied by stamped directed envelope.

**Notice to Advertisers and Subscribers.**

All Advertisements, Orders for Copies of The Hospital, and Business Communications should be addressed to The Publisher, *not to the Editor*, The Hospital, 140, Strand, W.C.

The Cost of Subscription, post free, is as follows:—Per week, 1½d.; per quarter, 1s. 8d.; per half-year, 3s. 3d.; per annum, 6s. 6d. Foreign:—Per annum, 8s. 8d.; payable, in all cases, in advance.

**NOTICE.—The Index for Vol. IX.. (ending March, 1891) is on sale at the Office of this Journal, price 2½d., post free.**

### GENERAL CHARITIES.

[This Column is devoted to an account of work of charitable institutions other than Medical Charities. The Editor will be glad to receive reports or news of work at 140, Strand. *Philanthropy* should be plainly written on the envelope.]

The **PLAYING FIELDS' COMMITTEE** have received substantial help in support of their useful, but probably rather up-hill, endeavours to provide new open spaces and preserve existing recreation grounds for the people of London, from the Grocers' Company and Goldsmiths' Company, who have each sent them a donation of £50, and by a donation of £21 from the Mercers'.

There was a large gathering at the summer fête of the **EARLSWOOD ASYLUM**, which took place last month in the grounds of the institution at Redhill. Much interest was shown in the competition for prizes in athletics by the children engaged in the various branches of industry conducted in the asylum, and in the exhibition of the various articles and models made by the pupils. The number of inmates, who come from all parts of the country, is now over 620.

The report of the Council of the **CENTRAL ASSOCIATION FOR DEALING WITH DISTRESS CAUSED BY MINING ACCIDENTS**, which was presented recently to the meeting at the Mansion House, shows a marked increase in the number of members. But notwithstanding this satisfactory feature the strain on the funds of the district associations continues to be very great. The number of disablement cases dealt with during the past year amounted to 39,411, and there were 2,396 widows and 3,842 orphans in receipt of annuities. The Chairman, Colonel Blundell, M.P., expressed a fear that the benefits had been calculated on too liberal a scale; as, though it is possible to measure approximately the probable number of accidents in the whole country, it is not so easy to forecast the number for any particular district. The desirability of adopting a system of re-insurance seems clear, and, as Lord Wharncliffe pointed out, the practice of paying away all the money received in relief is one of questionable policy. A society which has to deal with distress on so large a scale is in danger of getting into an unenviable state of debt if reserve funds are not built up.

Few, if any, districts of London will ere long be without their Technical and Recreative Institutes. One of the latest additions is situated in the Lewisham High Road, on the site of the old Royal Naval School. It is the handsome gift of the Goldsmiths' Company, who, at a cost of £280,000, have provided for the erection of the buildings, and the fitting, maintenance, and endowment of the Institute. It was originally intended that the expense of purchase and endowment should be shared with the Charity Commissioners, but the latter body of gentlemen were released by the company, doubtless much to their satisfaction, combined, we hope, with fitting gratitude, notwithstanding the fact that corporations are said to have no souls to afflict and no bodies to kick. The Goldsmiths' Company seem to have omitted nothing likely to contribute to the happiness, comfort, and even luxury of the inhabitants. The building and grounds contain, among other things, a library, the books of which have cost over £2,000, one of the finest and largest swimming baths in London, a magnificent gymnasium, reading-rooms, class-rooms for every conceivable branch of art, science, and literature, a bicycling and running track, and a cricket and football ground.

No unbiassed person will deny that the **OXFORD HOUSE** has contributed much to the elevation of the standard of morality and Church life in the East-end of London, and that the results achieved justify further endeavours in the same direction, not merely to promote Church work; more than that is wanted and is aimed at by the founders of the movement, which was started and has since been doing its work with an endeavour to meet all the reasonable wants of human nature. The attempt to grapple with the complicated social questions which have confronted us in the East-end, as well as in other districts of London which are at last attracting their share of attention, has met with far more success than was expected by many sceptical spirits. The present house, a disused national school, which was opened seven years ago, is no longer large enough for the requirements of the institution. Some misunderstanding seems to have arisen as to the object of the appeal for funds, much needed, for the completion of the new building. The extension is not required for young Oxford men to live in, but for the accommodation of the increasing number of those who are engaged in the different branches of work which have been organised by the House. In addition to this extension, it is intended to provide a club for 500 working men. Towards the cost of this £8,000 have already been contributed, but £4,000 more are still required. As Prebendary Eyton has pointed out, the comparative collapse of the People's Palace, which is of a very different character from the institution under notice, should not deter those who would be disposed to help the Oxford House. Active spirit and enthusiasm are no doubt of more value than bricks and mortar, but buildings are absolutely necessary for the carrying on of the work of such an institution as the Oxford House, and the appeal now made for funds is worthy of a liberal response. The fundamental idea of the movement is the spirit of self sacrifice, confraternity, and common life, and it is only by means of such a combination that we can hope to do permanent good and solve the great social problems that stare us in the face. When this and other kindred institutions were first established it was thought by many people that they were merely the outcome of hysterical emotion, and would soon be chronicled amongst things of the past. But the froth of emotion has subsided, and the elements which are permanent remain.

### SCRAPS AND GLEANINGS.

At the annual meeting of the Sunderland Infirmary it was proposed to erect a convalescent home in connection with the Infirmary.

At the monthly meeting of the Worcester Infirmary, on the 21st ult., it was decided to provide further accommodation for the nursing staff.

West Bromwich justly congratulates itself on having had a Hospital Saturday collection equivalent to Birmingham, at the small outlay of £73.

Good progress has been made during the year at the Sheffield Hospital, and an extension of the institution is contemplated in the immediate future.

At the annual meeting of the Governors of the Swindon Victoria Hospital, it was decided to expend the sum of £750 on extensions to the hospital.

A successful meeting of the Bury Friendly Societies on behalf of the Hospital Sunday Fund was held on a recent Sunday. A collection amounting to £27 was made in the church and in the streets.

The Clayton Hospital, Wakefield General Dispensary, and Leeds Infirmary, have all benefited by the Musical Festival held at Normanton on their behalf. The amount awarded to each institution was £33.

The Hospital Saturday collection in Brighton shows a total of £827 4s. 4d., against a total of £764 7s. from similar sources last year. A number of collecting sheets and sums from entertainments and other funds have yet to be received.

A very satisfactory report was presented at the annual meeting of the Warwick Cottage Hospital. Especial credit is due to the working population for the hearty manner in which they have contributed towards the expenses of the institution.

The Managing Committee of the Adelaide Hospital, Dublin, report that, in spite of an increase in the number of patients during the year, the accounts show a balance to credit. The Committee regret that they have so frequently to turn patients from the door.

The annual report of the Arbroath Infirmary shows that the Infirmary is in a very favourable condition. It was stated that during the two months during which the new Convalescent Home had been opened there had been numerous applications for admission.

An appeal is made for assistance to discharge the debt on the new All Saints Convalescent Hospital for Children at Eastbourne. This institution is of the greatest value, is restoring thousands of children to complete health, and is in every way worthy of support.

The lot of the pauper differs. Whilst the inhabitants of the Lambeth Workhouse were enjoying a strawberry feast at the public expense, a casual from Wallis Yard was receiving a sentence of four days' hard labour for protesting too energetically against a dole of sour bread.

At the annual meeting of the Liverpool Convalescent Institution, on the 18th inst., it was stated that the new building in connection with the Home was nearly completed. The report was of a highly satisfactory nature, and a balance to the good of £369 on the accounts was shown.

At the annual meeting of the Royal Derby and Derbyshire Nursing and Sanitary Association the Mayor of Derby stated that the Association had done an immense amount of good. The balance of £418 on that year's accounts was the largest recorded since the establishment of the institution.

At the half-yearly meeting of the Governors of the Essex and Colchester Hospital it was stated that the number of out-patients was exactly double that of the previous year, and that the number of in-patients had also considerably increased. The enlargement of the hospital will soon be imperative.

Through the energy of the Matron of the Weymouth Royal Hospital (Mrs. Parry), one of Cosens and Co.'s steamboats was chartered for a trip to Lulworth Cove. The Brunswick Band gave their services, and played a choice selection of music during the excursion. The proceeds went towards the funds of the hospital.

A very stormy meeting was held in Hertford on the 22nd ult., to consider the desirability of making Sunday collections for charitable institutions. "The better the day the better the deed" does not seem to be the opinion of many of the leading inhabitants of that town, and the subject of Sunday demonstrations is a vexed question.

The Hospital system of Dublin is the admiration of all who visit the metropolis. It appears that the total income of the Dublin hospitals receiving Government grants during the year ended in March last was £40,762, of which £15,722 was received from the Government, £3,606 subscriptions and donations, £3,517 bequests, and £5,949 interest on property.

The new Victoria Hospital for Poor Sick Children at Hull was opened on the 22nd inst. by the Marchioness of Salisbury. All the arrangements are very complete. One noticeable feature is a covered space for perambulators. It has been erected at a cost of £7,500 including furnishing. One thousand pounds is still required to free the institution from debt.

The proposed hospital for consumption and diseases of the chest at Belfast will probably be shortly commenced. The public have already subscribed £1,600 towards it, and Mr. Foster Green has made a donation of £2,000 on condition that the inhabitants of Belfast supplement this sum to an extent sufficient for the erection of a building containing not less than twenty beds.

The foundation-stone of the new wards of the Home for Sick Children at Cold Ash was laid on Tuesday by Miss Kitty Ricardo, daughter of Major and Mrs. Ricardo, of Donnington Castle House. The old wards, which are insufficient for accommodating the large number of sick children sent down from London, will be attached to the building by a covered corridor. There will be two wards, each containing eight beds, with nurses, sitting and bed rooms, affording accommodation for 22 inmates.

On the 29th ult., Mr. E. Brock, M.B., B.S.Lond., late Resident Medical Officer at the Royal Hospital for Diseases of the Chest, City Road, was presented on his resignation, by the nursing staff of that institution, with a very handsome walnut stationery-case; and by the patients with a fine inlaid oak plaque. In addition to this he received several private tokens of regard from the matron and sisters.

### THE STUDENT OF MEDICINE.

[All communications for this Supplement should be addressed to The Editor of The Hospital, at 140, Strand, with the words, "Students' Column," written in the left hand top corner of the envelope.]

### ON GERMAN STUDENTS (*continued*).

*The substance of a paper read before the St. Mary's Hospital Medical Society by* W. Overend, *B.A. Oxon.*

Passing on to the special subjects, such as Bacteriology, Ophthalmology, and Otology, these are taught with the same strictness of method as the general ones. For instance, in the eye department at Heidelberg, Becker, who died last year was a splendid teacher, but somewhat coarse and rough with the patients, and addicted to making more fun and ridicule of the phlegmatic students than ours would tolerate. The respect and veneration of the students for their professors is something unbounded—perhaps a little too servile. Every student in class stands up when he is spoken to, and remains so until told to resume his seat. Becker's courses were so excellent than an average man should certainly be thoroughly acquainted with the common diseases of the eye, but judging from the results it is evident that the German students are neither so sharp or so receptive as our own. They require line upon line and precept upon precept—perhaps they remember so much longer. The courses in bacteriology are usually held during the vacations lasting about four weeks. Their work begins at quarter past eight with a short lecture on the subject of the day, and finishing at five or six in the afternoon. Ernst, of Heidelberg, holds two courses annually in April and August. Commencing with the methods of bacteriological investigation and a refutation of former views as to the mutability of bacteriological species, we proceeded to the culture of organisms on potatoes and bread pulp. These food soils being previous sterilised. A set of glass apparatus was provided for each of two students at a cost of about 40s., the same amount being the fee for the course itself. During the first week we busied ourselves with the study of non-pathogenic bacteria, cultivation in hanging drops of water, the preparation of our gelatine and agar-agar mixtures filling a series of 100 or more test-tubes, which were sterilised before and after the introduction of these media. Among the organisms we studied were Bacteria megatherium, subtilis, proteus vulgaris, and zenkeri, B. cyanogenus of blue milk, and pyocyanous of green pus, B. prodigiosus, potato bacillus, mucor, Aspergillus, various sarcinæ, and the micro-organism of saliva. Several litres of the gelatine were prepared. This consisted of a cold infusion of meat with peptone, common salt, and gelatine. Frænkel's book was used as a textbook on the subject. The principles of the microscopic investigation of bacteria were then discussed. During the second week we made an examination of the micro-organisms of air, using Hesse's method and Esmarch's "roll-platten," also the micro-organisms of the Neckar water, the latter on gelatine plates, and then the bacilli of lactic fermentation. Afterwards we proceeded to the study of typhoid bacilli, the streptococci and staphylococci of pus, and the various forms of septicæmia—fowl fever, &c. The various methods of staining (Gram, Ehrlich, &c.) were mentioned, with elaborate instructions. The remainder of the course was devoted to anthrax and the staining of the spores, staining of bacteria in sputa, &c., and in sections. Five per cent. carbolic was mentioned as the best disinfectant for tubercular sputum, iodoform being absolutely powerless. We also had specimens of sperillum of Asiatic cholera, the fact being emphasized that it is only found between the epithelial cells and basement membrane of Lieberkühn's glands; and inoculation experiments in animals only succeed after the contents of the stomach have been neutralised and peristalsis paralysed by opium. The organism is found in all genuine cases of Asiatic cholera, and in no other disease. The students take one course at some time or other before their final examination. It is a little surprising to see that each possesses his own Zeiss and immersion lens, with Abbe's condenser, &c. When he is qualified, he is certainly able to examine sputa or urinary deposits for tubercle bacillus, to recognise gonococcus and prepare it, to examine pus, and his enthusiasm for the pathogenic army of cocci is certainly far beyond that of our own. With regard to the final examination: At the end of four and half years from his commencement, the student presents himself for the final examination or "Staats-examen," an examination which confers no title except that of "Praktischer Arzt" or qualified doctor. The scope of the examination is the same throughout Germany, and consists of ten subjects—Anatomy, Physiology, Pathology, Medicine, Surgery, Midwifery, Gynæcology, Pharmacology, Ophthalmology, and Insanity. In

The Hospital Advertiser.

THE STUDENT OF MEDICINE—(*continued*).

each subject there is a written, a practical, and a *viva voce*. Each subject is taken in a certain order and must be passed before going on to the next; the whole examination lasting perhaps five months. The examination in Pathology consists of paper work with questions on one or two subjects, a description of pathological specimens, and thirdly, and the most important, the making of a post-mortem and writing a description of the same. In Medicine there is also a paper on one or two subjects, then the student must examine a patient in the wards, make notes on the case, and write an essay during the course of the same day to be given to the Professor the next morning. This may be done at home. The case must be attended to daily for seven or eight days, and notes must be made recording its progress and treatment. Should the patient die in the meanwhile, post-mortem notes must be included. Surgery is somewhat similar with regard to patients, together with the performance of two or three operations on the dead body, and *viva voce* on instruments and bandaging.

### APPOINTMENTS.

At the Pendleton Branch Dispensary of the Salford Royal Hospital, John Valentine, M.B., C.M.Aberd., has been appointed house surgeon. At the Hospital for Consumption and Diseases of the Chest, Brompton, G. Hamilton Bristow, M.D., M.R.C.S., L.S.A., has been appointed house physician. At the Westminster Hospital, W. H. A. Tebbs, M.R.C.S., L.R.C.P., has been appointed house surgeon; A. M. Gossage, M.B.Oxon., and E. H. Sharman, M.R.C.S., L.R.C.P., house physicians. At the County Donegal Infirmary, Lifford S. Hamilton, M.D., M.A.O., has been appointed house surgeon. At the Rock Castle Colliery, Ammanford, Walter H. John, L.R.C.P., M.R.C.S., has been appointed medical officer. At Queen's College, Birmingham, J. F. Jordan, M.B., B.Ch.Irel., has been appointed demonstrator of anatomy. At the General Infirmary, Sheffield, W. S. Kerr, M.B., C.M. Edin., has been appointed third assistant house surgeon; H. T. Wightman, L.R.C.P., M.R.C.S., first assistant house surgeon, and Hugh Rodes, M.B., C.M., senior house surgeon. At the Oldham Infirmary, Richard McCraith, M.A., M.B., B.Ch. Dubl.Univ., has been appointed house surgeon. At the Edinburgh Royal Maternity and Simpson Memorial Hospital, T. F. Macdonald, M.B., C.M.Glas., has been appointed house surgeon, and W. T. Thomson, L.A.St. And., M.B., C.M.Edin., house surgeon. At the Aberdeen Royal Infirmary, J. Sckott Riddell, C.M., M.B., M.A.Aber., has been appointed assistant house surgeon.

### VACANCIES.

The following vacancies are announced this week, the amount of salary in each case being given after the name of the institution:

#### Resident Medical Officers.

Ancoats Hospital, Manchester, Resident Junior House Physician—Salary £50, and board, &c.
Cheshire County Asylum, Macclesfield, Junior Assistant Medical Officer Salary £125, with board, &c.
City Asylum, Birmingham, Resident Clinical Assistant—Board, &c.
Derbyshire Royal Infirmary, Resident Assistant House Surgeon—Salary £10 for first six months, £25 for second six months.
General Infirmary at Gloucester and the Gloucester Eye Institution, House Surgeon—Salary £10, with board, &c.
Hospital for Consumption and Diseases of the Chest, Brompton—House Physicians—Board, &c.
Liverpool Stanley Hospital, Junior House Surgeon—Salary £75, with board, &c.
Lunatic Hospital, The Coppice, Nottingham, Assistant Medical Officer—Salary £100, with board, &c.
Nottingham Borough Asylum, *locum tenens* for two months, from the middle of August.
Nottingham General Hospital, Resident Medical Assistants and Resident Surgical Assistants—Board, &c.
Royal Albert Hospital, Devonport, Assistant House Surgeon—Board.
Royal National Hospital for Consumption, Ventnor, Resident Medical Officer—Salary £100, with board, &c.
Stockport Infirmary, House Surgeon—Salary £100, with board, &c.
Western General Dispensary, Marylebone Road, N.W., Junior House Surgeon—Salary £50, with board, &c.
Wolverhampton and Staffordshire General Hospital, Wolverhampton, House Physician to act as Chloroformist, Pathologist, and Medical Registrar; also House Surgeon to act as Surgical Registrar—Salaries £100, with board, &c.

#### Non-Resident Medical Officers.

London Temperance Hospital, Hampstead Road, N.W., Registrar, Pathologist, and Anæsthetist—Salary 59 guineas.
Queen's College, Birmingham, Lecturer on Operative Surgery.
Society of Apothecaries of London, Secretary to the Medical Examiners and Secretary to the Board of Arts combined.

### The Hospital Advertiser.

### THE STUDENT OF MEDICINE—(*continued*).

### SCHOLARSHIPS AND PRIZES.

At Cavendish College, Cambridge, the following students have been recommended for election to scholarships in the recent open scholarship examination: J. H. Woolston, Wellingborough School, £50; A. E. Thorne, Rossall School £40; E. H. Vines, South-Eastern College, Ramsgate, £30; and M. W. Shute, Merchant Taylors' School, £30.

### PASS LISTS.

#### University of Aberdeen.

GRADUATION IN MEDICINE, JULY 29TH, 1891.

The following candidates received degrees in medicine and surgery:—

THE DEGREE OF M.D.—Arthur Stephen Inglis, M.B., C.M., Hastings; Alexander Nicoll, M.B., C.M., Queensland; Patrick John S. Nicoll, M.B., C.M., Ashton-under-Lyne.

THE DEGREES OF M.B. AND C.M.—John Ambrose, Aberdeen; Alexander Anderson, M.A., Edenkillie; Archibald C. Barron, Nairn; Robert Hugh G. Bruce, New Deer; John D. Burnett, Fraserburgh; Edgar H. Condon, India; James S. Cooper, New Machar; Sinclair Couper, Aberdeen; John G. Dallas, Nairn; William J. Devar, Arbroath; William Dunn, Tyrone; Hugh Fraser, M.A., Fearn, Ross-shire; Arthur Gardiner, Devonshire; Alexander Jamieson, Ellon; James Laird, Crathie; Donald Lamond, M.A., Crathie; John R. Levack, Aberdeen; John Leys, M.A., Crathie; Felix P. Maclennan, Lochcarron; Ernest C. Maguire, Aberdeen: James S. Mather, Friockheim; James Miller, Wick; Joseph E. Milne, M.A., Aberdeen; William J. Morton, N. S. Wales; Barrington William Mudd, Sussex; Harry O. Nicholson, Aberdeen; George Ogg, M.A., Newcastle-on-Tyne; William Paterson, Banchory; Thomas W. Philip, M.A., Skene; Ernest George S. Saunders, Devonshire; William T. Scott, M.A., Fyvie; George Skeen, Aberdeen; George M'I. C. Smith, M.A., Durham; Ellerington R. Turner, Sutherlandshire; James Walker, Stromness; James D. Walker, New Pitsligo; Robert A. Welsh, Cape Colony; George Wilson, M.A., Strichen; James S. Williamson, Auchmill.

John A. Gilchrist, Banchory, and James Mowat, Auchnagatt, have passed the examinations for the degrees of M.B. and C.M., but will not graduate until they attain the necessary age.

GRADUATION HONOURS.

The following gentlemen graduated with honours:—

HONOURABLE DISTINCTION.—Alexander Anderson, M.A., William J. Dewar, Hugh Fraser, M.A. John R. Levack, Ernest C. Maguire, Joseph E. Milne, M.A., William J. Morton, William T. Scott, M.A.

THE JOHN MURRAY MEDAL AND SCHOLARSHIP (awarded to the most distinguished graduate of the year).—William Findlay, M.A.

THE GEORGE THOMPSON FELLOWSHIP has been awarded to William Findlay, M.A.

THE DIPLOMA IN PUBLIC HEALTH has been conferred at this term on the following candidates: William H. Hewlett, M.B., C.M., Gloucester; Patrick B. H. M'Leod, M.B., C.M., New Deer; Irvine Kempt Reid, M.D., British Guiana.

DEGREES IN SCIENCE.

The degree of Bachelor of Science (B.Sc.) has been conferred on John Macdonald, M.A.

The following candidates have completed the first Bachelor of Science examination: Andrew T. Gage, M.A., Peter Mackessack.

RESULTS OF THE PROFESSIONAL EXAMINATIONS FOR MEDICAL DEGREES.

The following candidates have passed the First Division of the First Professional Examination for the degrees of M.B. and C.M.: J. H. Abrahams, John M. Andrew, Alex. Archibald, Ernest Barnes, Herbert Barraclough, James A. Black, Thomas I. Bonner, John H. I. Brown, George Bruce, R. F. Campbell, Wm. Cartwright, Alex. L. Cobban, Chas. G. Cowie, Robert Cruden, A. B. Cruickshank, Alex. Don, W. E. G. Duthie. George A. Gibb, John A. Gordon, George Grant, George C. Grant, Patrick Grant, Louis J. Helmrich, William C. Hossack, John G. Jones, W. M. Keith, Andrew D. Kelly, James S. Laing, V. van Langenberg, William Lethbridge, C. R. B. G. Lindsay, A. H. Lister, Alex. Low, Peter Macdonald, J. M. H. MacLeod, James Macpherson, John S Marr, Peter Mitchell, Edward Oliphant, C. E. F. Owen-Snow, A. F. Paterson, Edward M. Payne, Philip Prebble, Alex. Reid, George Reid, David Ross, Joseph H. Rowe, Alex. P. Rust, James L. Salmond, Robert Smith, George Thomson, William Thomson, George A. Troup, Robert Turner, W. S. O. Waring.

The following candidates have completed the first professional examination: J. W. S. Attygalle, G. G. M'L Black, G. W. Brown, G. A. Bruce, A. S. Cardno, L. Cartwright, Arnold T. Clarke, Henry G. L. Coates, L. O. Crosswell, J. C. T. Crowden, James B. Cruickshank, D. H. Dantra, John Low Dickie, F. P. Elliot, J. L. G. Gillanders, J. H Goodliffe, Peter Harper, H. G. Hoad, Robert Johnstone, C. E. Maguire, James Mansie, Edward Proudlove, Alex. W. Reid, John Russell, James Smith, *Alex. Stables, John G. Stuart, Robert S Trotter.

The following candidates have passed the Second Professional Examination: Alex. Anderson, Andrew Baxter, William Boddie, James Broomhead, Albert Brown, John Cameron, David Crichton, N. B. Darabsheth, Andrew Davidson, C. M B Dawson, W. C. Duthie, Robert Ferguson, Duncan Finlayson, Alex. Forbes, Gordon Fowler, *James Gillespie, William Grant, E. M. Griffith, Alexander Innes, W. A. Justice Thomas B. Law, John Leach, John R. May, Joseph E. Milne, Henry M'C. Mitchell, William Moir, *Thomas W. Ogilvie, Mark Polson, James Ritchie, W. A. G. Russell, Bertram Saunders, William H. de Silva, Charles Simpson, William Sinclair, A. W. Thomson, *William Trethowan.

* Indicates that the candidate has passed "with credit."
** Indicates that the candidate has passed "with much credit."

### The Hospital Advertiser.

### THE STUDENT OF MEDICINE—(*continued*).

### Royal College of Physicians of London.

The following gentlemen, having passed the required examination, have been admitted Members of the College: Winslow Anderson, M.D. California, Portman Mansions; George Bainbridge, L.R.C.P., Indian Medical Service; Roland Danvers Brinton, M.D. Camb., Queen's Gate Terrace; Frederick William Burton-Fanning, M.D. Camb., Norwich; Frederick Foord Caiger, M.D. Lond., South-Western Hospital; John Elliott, M.D. Lond., Chester; William Soltau Fenwick, M.D. Lond., Consumption Hospital; Herbert-Morley Fletcher, M.B. Camb., St. Bartholomew's Hospital; Anghel Gaster, M.D. Bucharest, Warwick Road; Frank Papillon Haviland, M.B. Camb., University Club; James Samuel Risien Russell, M.B. Edin., Wimpole Street; Frederic La Coque Thorne, M.D. St. And., Leamington Spa; William Bezly Thorne, M.D., St. And., Gledhow Gardens; Stuart Alexander Tidey, M.B. Lond., Montreux; Hugh Walsham, M.B. Camb., Cromwell Road; William Alfred Wills, M.D. Lond., Davies Street.

### University of Glasgow.

The following candidates have passed the Fourth (Final) Professional Examination for the degrees of M.B. and C.M.:—

Candidates who Passed in Pathology in Third Professional Examination.—William Maclachlan Allardice, William Anderson, Jas. Gordon Bain, Peter Otho Watkin Browne, David Coutts, John Frew, Hugh Galt, Edwin Arthur Gibson, John Gilmour, David Wyllie Girvan, John Angus Green, Walter Buchanan Hastings, Robert Home Henderson, Robert Robertson Kilpatrick, Peter Alexander Laird, David Lamb, John William Logie, Robert Mortimer Malcolm, Ernest Louis Marsh, Thomas Milbourne Martin, John Kerr Muir, William Murray, Donald Neil Macfarlane, Robert M'Ghie, John M'Gregor, Charles Hugh M'Ilwraith, M.A.; Daniel M'Kenzie, Percy George M'Reddie, Robert William Nairn, Peter Paterson, Robert Sharp, David Smith, George Clark Stewart, Robert Stobo, George Mervyn Sydenham, James Todd, Ebenezer Turner, William Watson, James Laurie White, James William White, James Allan Wilson, Robert Wilson, William Young, John Yuill.

Candidates who took Pathology in Fourth Professional Examination.—James Aitken, James Alexander Aitken, John Fulton Barr, B.Sc., John Adam Boyd, Alexander Campbell, Niel Campbell, David Christie, Samuel Cockburn, John Alexander Creighton, William Crichton, John David, Hugh Colligan Donald, William Doyle, Jas. Gray Duncanson, Alfred Ernest Evans, William Bertram Ochiltree Ferguson, Andrew Goldie, Richard Hamilton, Yousuf Hamis, John Atkinson Harrison, Anthony Inglis, William Jackson, Robt. James, John Harris Jones, Owen Gethin Jones, Charles Lavery, David Lloyd, Matthew Lochhead, B.Sc., James Marr, William Mason, John Wilson Mathie, John Miller, Robert Walker Moir, David Muir, Dugald Macdougall, Neil Macintyre, William James M'Kendrick, B.Sc., Alexander Lewis M'Leod, M.A., James Henderson Naismith, Gilbert Park, Hugh Hamilton Park, William Park (Beith), Robert Alexander Paton, Brownlow Riddell, Andrew Robertson, Robert Currie Robertson, M.A., John Gilfillan Ronald, Wm. David Rose, Archibald George Sanders, Arthur Thomas Scott, M.A., Charles Symington, Daniel Macpherson Taylor, M.A., Lewis Dunbar Temple, William Thompson Merry Wallis, David Watson, Martin Whyte.

### Victoria University.

Faculty of Medicine.

The following gentlemen have satisfied the Examiners:—

First Examination (Part II.).—Biology: T. W. Arnison, Owens College; S. R. Christophers, University College; Harold Coates, A. V. Davies, J. L. Gardner, Alfred Greenwood, and Harry Homer, Owens College; John Hay, University College; J. B. Holmes and W. F. Jackson, Owens College; R. B. Jones, University College; C. E. Ligertwood, Yorkshire College; T. H. Miller, Owens College; F. W. Morris, University College; Newman Neild, Owens College; P. Nelson, University College; W. A. Newall, G. S. Hickerson, and Thomas O'Neill, Owens College; C. J. Palmer and C. J. L. Palmer, University College; J. H. Renshaw, Owens College; J. V. Shaw, E. A. Smith, R. H. Trotter, and H. de C. Woodcock, Yorkshire College.

Second Examination.—First Division: J. H. Crocker and Alan McDougall, Owens College; H. T. Nixon, University College. Second Division: J. H. Ashworth, Owens College; R. E. Bickerton, University College; H. A. Bold, Owens College; D. E. Darbyshire and F. H. Day, University College; W. D. Hayward, Owens College; E. H. L. Lindon, University College; V. E. H. Lindsay, Owens College; E. D. Minshall, University College; M. R. Rhodes, Owens College; H. A. Robinson, University College; C. F. Thompson, Yorkshire College.

Final Examination (Part I.).—W. E. Barker, Owens College; Arthur Bicknell, University College; J. C. Buckley, F. J. H. Coutts, J. L. B. Dixson, and C. C. Garfit, Owens College; J. H. Lightbody, University College; C. E. M. Lowe, E. C. McCarthy, C. R. Marshall, Alfred Murgatroyd, C. H. G. Ramsbottom, C. B. Taylor, R. T. Turner, and W. A. Wilkinson, Owens College.

Distinguished in Pharmacology and Therapeutics.—F. J. H. Coutts and C. R. Marshall, Owens College.

Distinguished in Obstetrics.—E. C. McCarthy and C. R. Marshall, Owens College.

Final Examination (Part II.).—First Division: Samuel Crawshaw and W. B. Warrington, Owens College. Second Division: Alfred Harris, University College; G. F. Jones, Owens College; S. R. Knight, University College; H. K. Ramsden and W. C. Rigby, Owens College; J. H. Shaw and A. C. Wilson, University College.

Examination for Diplomas in Sanitary Science.—N. F. H. Fitzmaurice, Andrew Gray, W. H. Hewlett, T. J. Monaghan, W. D. Prendergast, E. A. Rawson.

